# Predictors for Prolonged Hospital Stay Solely to Complete Intravenous Antifungal Treatment in Patients with Candidemia: Results from the ECMM *Candida* III Multinational European Observational Cohort Study

**DOI:** 10.1007/s11046-023-00776-4

**Published:** 2023-08-11

**Authors:** Matthias Egger, Jon Salmanton-García, Aleksandra Barac, Jean-Pierre Gangneux, Hélène Guegan, Valentina Arsic-Arsenijevic, Tadeja Matos, Rok Tomazin, Nikolai Klimko, Matteo Bassetti, Helena Hammarström, Eelco F. J. Meijer, Jacques F. Meis, Juergen Prattes, Robert Krause, Oguz Resat Sipahi, Ulrike Scharmann, P. Lewis White, Guillaume Desoubeaux, Julio García-Rodríguez, Carolina Garcia-Vidal, Sonia Martín-Pérez, Maite Ruiz, Mario Tumbarello, Alida Fe Talento, Benedict Rogers, Katrien Lagrou, Jens van Praet, Sevtap Arikan-Akdagli, Maiken C. Arendrup, Philipp Koehler, Oliver A. Cornely, Martin Hoenigl, on behalf of the ECMM Candida III Study Group$, Ana Alastruey-Izquierdo, Nick Alexander de Jonge, Tihana Bicanic, Ola Blennow, Blandine Denis, Nina Khanna, Cornelia Lass-Flörl, Clare Logan, Laura Loughlin, Volkan Özenci, Zdenek Zdenek, Laman Rahimli, Riina Rautemaa-Richardson, Joerg Steinmann, Igor Stoma, Janina Trauth, François Danion, Jochem B. Buil, Julio Dávila-Valls, Eric van Wijngaerden

**Affiliations:** 1 Division of Infectious Diseases, Department of Internal Medicine, Medical University of Graz, Auenbruggerplatz 15, 8036 Graz, Austria; 2 Biotech Med, Graz, Austria; 3 Translational Medical Mycology Research Unit, ECMM Excellence Center for Medical Mycology, Medical University of Graz, Graz, Austria; 4 Faculty of Medicine and University Hospital Cologne, Institute of Translational Research, Cologne Excellence Cluster on Cellular Stress Responses in Aging-Associated Diseases, University of Cologne, Cologne, Germany; 5 Department I of Internal Medicine, Faculty of Medicine and University Hospital Cologne, Excellence Center for Medical Mycology, University of Cologne, Cologne, Germany; 6 Clinic for Infectious and Tropical Diseases, University Clinical Center of Serbia, Faculty of Medicine, University of Belgrade, Belgrade, Serbia; 7 CHU Rennes, Inserm, EHESP, Irset (Institut de Recherche en santé, environnement et travail), Univ Rennes, UMR_S 1085, 35000 Rennes, France; 8 Faculty of Medicine, Institute of Microbiology and Immunology, Medical Mycology Reference Laboratory (MMRL), University of Belgrade, Belgrade, Serbia; 9 Centre for Microbiology, Institute of Public Health of Vojvodina, Novi Sad, Serbia; 10 Institute of Microbiology and Immunology, Faculty of Medicine, University of Ljubljana, Ljubljana, Slovenia; 11 Department of Clinical Mycology, Allergy and Immunology, North Western State Medical University, St Petersburg, Russia; 12 Department of Health Sciences (DISSAL), University of Genoa, Genoa, Italy; 13 IRCCS Ospedale Policlinico San Martino, Infectious Diseases Unit, Genoa, Italy; 14 Department of Infectious Diseases, Institute of Biomedicine, Sahlgrenska Academy, University of Gothenburg, Gothenburg, Sweden; 15 Medical Microbiology and Infectious Diseases, Canisius-Wilhelmina Hospital (CWZ), Nijmegen, the Netherlands; 16 Center of Expertise for Mycology Radboudumc-CWZ, Nijmegen, the Netherlands; 17 Department of Medical Microbiology, Radboud University Medical Center, Nijmegen, The Netherlands; 18 Department of Infectious Diseases and Clinical Microbiology, Ege University Faculty of Medicine, Izmir, Turkey; 19 Institute of Medical Microbiology, University Hospital Essen, University of Duisburg-Essen, Essen, Germany; 20 Public Health Wales, Center for Trials Research/Division of Infection/Immunity, Microbiology Cardiff and Cardiff University, Cardiff, UK; 21 Department of Parasitology-Mycology-Tropical Medicine, CHRU de Tours, Tours, France; 22 Microbiology Department, La Paz University Hospital, Madrid, Spain; 23 Department of Infectious Diseases, Hospital Clinic de Barcelona, Barcelona, Spain; 24 Hospital Nuestra Señora de Sonsoles, Ávila, Spain; 25 Unit of Infectious Diseases and Microbiology, Institute of Biomedicine of Seville, University Hospital Virgen del Rocio, Seville, Spain; 26 Centro de Investigación Biomédica en Red de Enfermedades Infecciosas, Madrid, Spain; 27 Fondazione Policlinico A. Gemelli IRCCS, Rome, Italy; 28 Department of Microbiology, Beaumont Hospital, Dublin, Ireland; 29 Department of Clinical Microbiology, University Hospitals of Leicester NHS Trust, Leicester, UK; 30 Department of Microbiology, Immunology and Transplantation, KU Leuven, Louvain, Belgium; 31 Department of Laboratory Medicine, and National Reference Centre for Mycosis, University Hospitals Leuven, Louvain, Belgium; 32 Nephrology and Infectious Diseases, AZ Sint-Jan Brugge Oostende AV, Brugge, Belgium; 33 Department of Medical Microbiology, Hacettepe University Medical School, Ankara, Turkey; 34 Unit of Mycology, Statens Serum Institut, Copenhagen, Denmark; 35 Department of Clinical Microbiology, Rigshospitalet, Copenhagen, Denmark; 36 Department of Clinical Medicine, University of Copenhagen, Copenhagen, Denmark; 37 German Centre for Infection Research (DZIF), Partner Site Bonn-Cologne, Cologne, Germany; 38 Faculty of Medicine and University Hospital Cologne, Clinical Trials Centre Cologne (ZKS Köln), University of Cologne, Cologne, Germany

**Keywords:** *Candida tropicalis*, *Candida auris*, *Candida albicans*, *Candida parapsilosis*, *Candida glabrata*, Mortality, Guidelines

## Abstract

**Background:**

To date, azoles represent the only viable option for oral treatment of invasive *Candida* infections, while rates of azole resistance among non-albicans *Candida* spp. continue to increase. The objective of this sub-analysis of the European multicenter observational cohort study Candida III was to describe demographical and clinical characteristics of the cohort requiring prolonged hospitalization solely to complete intravenous (iv) antifungal treatment (AF Tx).

**Methods:**

Each participating hospital (number of eligible hospitals per country determined by population size) included the first ~ 10 blood culture proven adult candidemia cases occurring consecutively after July 1st, 2018, and treating physicians answered the question on whether hospital stay was prolonged only for completion of intravenous antifungal therapy. Descriptive analyses as well as binary logistic regression was used to assess for predictors of prolonged hospitalization solely to complete iv AF Tx.

**Findings:**

Hospital stay was prolonged solely for the completion of iv AF Tx in 16% (100/621) of candidemia cases by a median of 16 days (IQR 8 – 28). In the multivariable model, initial echinocandin treatment was a positive predictor for prolonged hospitalization to complete iv AF Tx (aOR 2.87, 95% CI 1.55 – 5.32, *p* < 0.001), while (i) neutropenia, (ii) intensive care unit admission, (iii) catheter related candidemia, (iv) total parenteral nutrition, and (v) *C. parapsilosis* as causative pathogen were found to be negative predictors (aOR 0.22 – 0.45; *p* < 0.03).

**Interpretation:**

Hospital stays were prolonged due to need of iv AF Tx in 16% of patients with candidemia. Those patients were more likely to receive echinocandins as initial treatment and were less severely ill and less likely infected with *C. parapsilosis*.

## Introduction

Invasive candidiasis (IC) including candidemia remains the most frequent invasive fungal infection in European and worldwide hospitals [1, 2]. Mortality rates are high with around 37% expected to have a fatal outcome at day 30 [3, 4]. Early adequate antifungal treatment is efficacious and one of the most important predictors of survival [5]. The European Society for Clinical Microbiology and Infectious Diseases (ESCMID) [6] and the Infectious Diseases Society of America (IDSA) guidelines [7] recommend echinocandins as first line treatment, which can be administered intravenously only. While stepdown to oral fluconazole treatment can be considered after 5 days or even earlier [8, 9], fluconazole has a narrower spectrum of activity paired with the continuous emergence of azole resistance among non-albicans *Candida* spp., including *C. glabrata *[10], is less well tolerated than echinocandins causing hepatotoxicity, and also lacks activity against biofilms [11]. Therefore, stepdown to an oral agent is not always feasible and prolonged intravenous (iv) antifungal treatment (AF Tx) may be necessary. This may prevent otherwise timely discharge of patients, meaning prolonged hospitalization to complete iv AF Tx or daily outpatient iv treatment, if available. Results are increased costs, risk for nosocomial infections, and avoidable waste of resources. New antifungals, including rezafungin (echinocandin with prolonged half-life allowing once-a-week administration) and ibrexafungerp (oral triterpenoid glucan synthase inhibitor) are currently in late stage clinical development and may help tackle this problem [12, 13]. To utilize these new drugs most efficaciously, studies are needed to identify patient populations that would benefit most from these new antifungal treatments. However, demographic and clinical factors that are associated with prolonged hospitalization solely to complete iv AF Tx have to date not been sufficiently described.

In order to delineate characteristics, as well as identify predictors for prolonged hospitalization solely to complete AF Tx, we performed a sub-analysis of the ECMM Candida III dataset [4].

## Methods

### Study Design and Participating Centers

The ECMM designed and conducted the CANDIDA III study—its third pan European multicenter observational cohort study over the past 25 years [4, 14, 15]—to collect data on epidemiology, risk factors, treatment, and outcomes of culture proven candidemia across Europe, as well as to assess the impact of guideline adherence on survival [4]. This represents a sub-analysis of CANDIDA III [4], where each participating hospital included the first ~ 10 blood culture proven adult candidemia cases occurring consecutively after July 1^st^, 2018. In short, participating centres/treating clinicians entered data on patient demographics, risk factors and characteristics, duration of hospitalization, diagnostic procedures, causative *Candida* species, treatment characteristics including antifungal treatment, whether hospital stay was prolonged only for completion of parenteral antifungal treatment, and outcomes, into the ECMM *Candida* Registry—*Candi*Reg – FungiScope® (NCT 01731353) [16, 17], on www.clinicalsurveys.net (EFS Fall 2018 Questback, Cologne, Germany) [4]. Candidemia was defined as the isolation of *Candida* species from blood culture, and the subanalysis was performed within the pool of those 621 where *Candida* spp. was reported. The main objective of this sub-analysis was to describe demographic and clinical characteristics of the cohort requiring prolonged hospitalization to complete iv AF Tx.

### Statistical Analysis and Ethics

For patients with prolonged hospitalization solely to complete iv AF Tx descriptive statistical analysis was performed for clinical characteristics and demographic variables, as well as for the distribution of *Candida* species. Case level data is available from corresponding authors by request. Data were summarized employing frequencies, percentages, mean and standard deviation (SD) or median and interquartile range (IQR) as appropriate. Binary logistic regression was used to assess for predictors of prolonged hospitalization solely to complete iv AF Tx in 30-day candidemia survivors. The reason for focusing only on 30-day survivors was to avoid bias that could have been introduced by including patients who had a fatal outcome before they could even possibly meet the event definitions of prolonged hospitalization solely to complete iv AF Tx. First, univariable analysis of clinical predictors was performed. All variables significant at *p* ≤ 0.15 in univariable analyses were considered as possible predictor variables for multivariable analysis.

This study was performed in accordance with the ethical standards laid down in the 1964 Declaration of Helsinki and its later amendments. For the database, retrospective data entry, and data analysis a central ethical approval was obtained at the University of Cologne, Germany (EK 17–485) that indicates that, generally, neither informed consent nor institutional review board (IRB) approval of each participating hospital would be required. Each participating hospital was required to obtain local IRB confirmation or approval as deemed necessary by the respective local regulations/authorities. Two-sided *p* < 0.05 was taken as cut-off for statistical significance. All statistical analyses were performed using IBM SPSS Statistics 25 (SPSS Inc., Chicago, IL, USA) and R (version 4.3.1; www.r-project.org).

## Results

A total of 621 unique patients with culture proven candidemia were included in the parent study. Of those, 16.1% (100/621), reported from 33 institutions in 16 countries had prolonged hospitalization specifically for the completion of iv AF Tx according to the treating physician. Hospitalization was prolonged solely to complete iv AF Tx by a median of 16 days (IQR 8 – 28; mean 22 days, SD 19 days; data available from 81 patients); the total duration of hospitalization in that group was median 21 days (IQR 15–45, mean 35 days). The median duration of hospitalization in those without prolonged hospitalization for iv AF Tx (n = 521) was 14 days (IQR 5–30 days; mean 22 days, SD 42).

For the cohort of 100 patients with prolonged hospitalization solely to complete iv AF Tx, patient characteristics, risk factors, treatment, as well as distribution of *Candida* spp. are displayed in Table 1. The majority (62%) were male and mean age was 57.6 years (SD 15.4). The most common underlying conditions were hematological/oncological malignancy (32%), diabetes mellitus (type I and II, 18%), ICU admission (17%) and major surgery (15%). Candidemia was classified as catheter related bloodstream infection (CRBSI) in 8% of those cases. *C. albicans* was the most common causative pathogen (42%), followed by *C. glabrata* (31%), *C. parapsilosis* and *C. tropicalis (*6% each), *C. krusei* (4%) and *C. auris* (3%), and other rare *Candida* spp. (4%).

**Table 1:** Patient characteristics and distribution of *Candida* species in patients with prolonged hospital stay solely to complete intravenous (iv) antifungal treatment (AF Tx) and in those without (excluding patients who received ambulatory parenteral treatment)

	Patients with prolonged hospital stay solely to complete iv AF Tx (*N *= 100) (%)	Other patients without prolonged hospital stay solely to complete iv AF Tx and without ambulatory parenteral antifungal treatment (*N* = 494) (%)
Age, mean (SD), y	57.6 (15.4)	63.1 (15.1)
*Sex – no. (%)*		
Male	62 (62%)	280 (57%)
*Risk factors for IC*		
SOT	3 (3%)	10 (2%)
BMI ≥ 30	15 (15%)	84 (17%)
Diabetes mellitus	18 (18%)	115 (23%)
Prosthetic valves/ other foreign body	15 (15%)	63 (13%)
Total parenteral nutrition	8 (8%)	122 (25%)
Major surgery	15 (15%)	138 (28%)
ICU	17 (17%)	203 (41%)
ECMO	1 (1%)	14 (3%)
Haematological/Oncological malignancy	32 (32%)	203 (41%)
*Countries*		
Serbia	25 (25%)	4 (1%)
France	19 (19%)	46 (9%)
Spain	8 (8%)	37 (8%)
Germany	6 (6%)	63 (13%)
Slovenia	6 (6%)	4 (1%)
Russia	6 (6%)	25 (5%)
United Kingdom	5 (5%)	87 (18%)
Italy	5 (5%)	24 (5%)
Austria	4 (4%)	26 (5%)
Netherlands	4 (4%)	19 (4%)
Sweden	4 (4%)	14 (3%)
Turkey	4 (4%)	61 (12%)
Others	4 (4%)	84 (17%)
Causative Candida species*		
*C. albicans*	42 (42%)	235 (48%)
*C. glabrata*	31 (31%)	92 (19%)
*C. parapsilosis*	6 (6%)	75 (15%)
*C. tropicalis*	6 (6%)	38 (8%)
*C. krusei*	4 (4%)	12 (2%)
*C. auris*	3 (3%)	11 (2%)
*C. dubliniensis*	1 (1%)	9 (2%)
*C. lusitaniae*	1 (1%)	4 (1%)
*C. rugosa*	1 (1%)	2 (0.4%)
*C. digboiensis*	1 (1%)	0
Others	0	22 (4%)
Not specified	4 (4%)	11 (2%)
Isolate susceptible to fluconazole°	53 (53%)	–
Isolate intermediate to fluconazole°	13 (13%)	–
Isolate resistant to fluconazole°	18 (18%)	–
Clinical course and outcome		
CRBSI	8 (8)	116 (24%)
Charlson Comorbidity Index, median (IQR)	5 (3–8)	5 (3–8)
Complications*		
Cardiac involvement	7/55 (13%)	16/157 (10%)
Eye involvement	5/42 (12%)	14/116 (13%)
Mixed fungal infections	5 (5%)	29 (6%)
ID/Microbiology Treatment Consultation	85 (85%)	391 (89%)
Treatment		
Started echinocandin	73 (73%)	274 (56%)
Started fluconazole and switched to echinocandin later	5 (5%)	–
Started voriconazole and switched to LAmB	1 (1%)	–
Started LAmB	2 (2%)	–
Other/unknown	19 (19%)	–
Treatment for ≥ 14 days after last positive blood culture	72 (72%)	229 (47%)
Days prolonged hospital stay for completing iv AF Tx, median (IQR)	16 (8–18)	0
30-day mortality	10/100 (10%)	219 (44%)
Overall mortality	19/100 (19%)	261 (53%)

IC = invasive candidiasis, SOT = solid organ transplantation, BMI = body mass index, ICU = intensive care unit, ECMO = extracorporeal membrane oxygenation, CRBSI = catheter related bloodstream infection, ID = infectious diseases, LAmB = liposomal amphotericin B, IQR = interquartile range

*Echocardiography performed in 55 patients; Ophthalmoscopy performed in 42 patients with prolonged hospital stay solely to complete iv AF Tx.

°Based on local susceptibility classifications.

In about half the cases (55%) echocardiography was performed, showing cardiac involvement in 13% of those examined. Eye exam was reported in 42% of cases showing ocular involvement in 12%. The vast majority (85%) received treatment consultation by an infectious diseases and/or microbiology expert. All-cause mortality at day 30 was 10%, and in 6/10 cases with a fatal outcome by day 30 investigators classified the death as unrelated to candidemia.

Echinocandins were the first line antifungal drug in 73% of cases. Another 5% were started on fluconazole but were then switched to an echinocandin, while 3% were either started on or switched to liposomal amphotericin B (Table 1).

Informed by univariable binary logistic regression, we evaluated predictors for prolonged hospitalization solely to complete iv AF Tx in patients who were alive at day 30 after diagnosis (*n* = 383; 30-day mortality in the overall cohort was 38%). Indicators for patients who were severely ill [= intensive care unit (ICU) admission, catheter related bloodstream infections (CRBSI), major surgery, neutropenia, total parenteral nutrition (TPN)] showed negative odds ratios (OR) for prolonged hospitalization solely to complete iv AF Tx (OR 0.22 – 0.48; *p* =  < 0.001 – 0.022). Concerning causative *Candida* species, patients with *C. parapsilosis* candidemia were less likely (OR 0.26, 95% CI 0.009 – 0.75, *p* = 0.012) to experience prolonged hospitalization solely to complete iv AF Tx in contrast to patients with *C. glabrata* candidemia (OR 1.90, 95% CI 1.11 – 3.25, *p* = 0.019). In the multivariable model, neutropenia, ICU admission, CRBSI, TPN, and *C. parapsilosis* remained significant negative predictors for prolonged hospitalization solely to complete iv AF Tx, while initial echinocandin treatment (aOR 2.87, 95%CI 1.55 – 5.32, *p* < 0.001) remained a strong positive predictor. In contrast, *C. glabrata* as causative pathogen failed to reach statistical significance (aOR 1.74 95% CI 0.95 – 3.20, *p* = 0.075) (Table 2).

**Table 2:** Univariable and multivariable binary logistic regression models for predictors of prolonged hospitalization solely to complete intravenous (iv) antifungal therapy (AF Tx) compared to patients without prolonged hospitalization to complete iv AF Tx in 30 day survivors (n = 383)

Variable	Univariable odds ratio	95% CI	*p*-value
*Demographics*			
Male, Sex	1.32	0.80–2.19	0.281
Advanced Age (per 10 years)	0.79	0.58–1.06	0.111
*Coexisting conditions*			
BMI ≥ 30	1.00	0.53–1.92	0.989
Solid organ transplantation	1.90	0.46–7.76	0.373
*Haematological/oncological*			
malignancy	0.68	0.40–1.14	0.144
Neutropenia (< 500/µL)	0.35	0.18–0.70	0.002
*Major surgery including abdominal*			
surgery	0.47	0.25–0.90	0.022
Diabetes mellitus (type I or II)	0.93	0.51–1.70	0.817
*Clinical factors*			
Intensive care unit admission	0.48	0.27–0.86	0.013
Catheter related bloodstream infection	0.22	0.09–0.52	< 0.001
Extracorporeal membrane oxygenation	0.53	0.06–4.34	0.552
TPN	0.31	0.14–0.67	0.003
Charlson Comorbidity Index	0.96	0.89–1.05	0.376
*Candida* spp. (n)°			
*C. albicans (165)*	0.94	0.57–1.55	0.821
*C. glabrata (90)*	1.90	1.11–3.25	0.019
*C. parapsilosis (54)*	0.26	0.09–0.75	0.012
*C. tropicalis (20)*	0.64	0.18–2.26	0.492
*C. krusei (9)*	1.07	0.22–5.24	0.936
*C. auris (9)*	1.90	0.46–7.76	0.373
*C. dubliniensis (8)*	0.53	0.06–4.34	0.552
Other *Candida* Species (10)*	1.01	0.97–1.06	0.491
*Clinical course (i.e., not baseline variables)*			
Mixed fungal infections	1.00	0.57–1.76	0.992
Infection consultation (ID or microbiology)	1.41	0.68–2.92	0.356
*Treatment*			
Initial echinocandin treatment	2.61	1.47–4.62	< 0.001
Stepdown to fluconazole	1.30	0.79–2.14	0.299

Variables	Multivariable odds ratio	95% CI	*p*-value
Age	0.78	0.56–1.08	0.131
Hematological malignancy	1.42	0.64–3.12	0.389
Neutropenia	0.25	0.09–0.67	0.006
Major surgery	0.62	0.31–1.27	0.192
Intensive care unit	0.45	0.24–0.85	0.014
Catheter related bloodstream infection	0.22	0.09–0.56	0.002
Total parenteral nutrition	0.31	0.13–0.70	0.005
*C. glabrata*	1.74	0.95–3.20	0.075
*C. parapsilosis*	0.28	0.09–0.85	0.025
Initial echinocandin treatment	2.87	1.55–5.32	< 0.001

°Candida species not specified (*n* = 18).

*Others include: C. rugosa (*n* = 3), C. pelliculosa (*n*  = 2), C. lusitaniae (*n* = 2), C. inconspicua (*n* = 1), C. guilliermondi (*n*  = 1), and C. digboiensis (*n*  = 1).

In addition, 4.3% (27/621) of patients included in the parent study from 16 institutions (including three institutions from France and two from Belgium, Germany, Netherlands, Spain and United Kingdom each) received outpatient intravenous antifungal therapy (all did not have prolonged hospital stay for completion of iv AF Tx). Among those, *C. albicans* and *C. glabrata* were the most frequent pathogens (each 38%; 10/26), followed by *C. tropicalis*, *C. auris* and *C. parapsilosis* (each 8%; 2/26). 30-day mortality was 11% (3/27) and overall mortality 22% (6/27).

## Discussion

We performed a sub-analysis of a multicenter observational study of candidemia, involving 64 hospitals from 20 countries across Europe, and found that 16.1% of patients with candidemia had prolonged hospitalization solely to complete iv AF Tx. These were less severely ill, had higher survival rates [4] and were less likely infected with *C. parapsilosis*. Outpatient iv AF Tx was performed in an additional 4.3% of the study population, outlining that in 20.4% no (sufficient) oral treatment options were available.

Our study highlights that there was a significant group of patients where step-down to oral fluconazole [8] was not performed. In the multivariable model, indicators of severe disease, namely neutropenia, ICU admission and TPN, but also CRBSI and *C. parapsilosis* as a causative pathogen remained significant negative predictors for prolonged hospitalization solely to complete iv AF Tx, likely because some of these factors were per se associated with longer duration of hospitalization. In contrast, initial echinocandin treatment and candidemia caused by *C. glabrata* were positive predictors for prolonged hospitalization solely to complete iv AF Tx, with the latter not reaching statistical significance in the multivariate model. However, *C. glabrata* was even more overrepresented among the smaller group of 27 patients who received outpatient iv AF Tx, of whom 38% had candidemia caused by *C. glabrata*, underpinning the impact of this organism that often shows resistance to azoles. The fact that *C. parapsilosis*, a pathogen associated with lower mortality and often causing CRBSI [10], was a negative predictor for prolonged hospitalization solely to complete iv AF Tx, indicates that this pathogen is still often (deemed) susceptible to oral azoles in Europe. Of note, azole resistance is also increasing among *C. parapsilosis* with several outbreaks reported [18, 19]. Therefore, lack of reliable oral treatment options may also depict a future problem for infections caused by *C. parapsilosis*.

In our cohort 30-day all-cause mortality was 10% (11% in those with outpatient iv AF Tx) compared to 44% in the remaining Candida III cohort without prolonged hospitalization solely to complete iv AF Tx or outpatient iv AF Tx (including patients who did not survive long enough to receive prolonged therapy), outlining the better prognosis and less severe disease course [4].

Importantly, initial echinocandin therapy was not only a positive predictor for prolonged hospitalization solely to complete iv AF Tx, but was also associated with better outcomes and survival in the overall study cohort [4]. More and better options for antifungal treatment outside the hospital will emerge in the near future, with a promising antifungal pipeline [20]. Rezafungin, an echinocandin with improved penetration into the peritoneal fluid and prolonged half-life, allowing once weekly infusion, has shown non-inferiority to caspofungin for treatment of IC in a landmark phase III trial [21]. Rezafungin will also be a viable option for patients that are incapable of taking oral medication. Ibrexafungerp [22], representing a novel antifungal class with an echinocandin like mechanism of action and excellent oral bioavailability [12], is FDA approved for vulvovaginal candidiasis and has shown promising results for IC in an ongoing phase IIb study [23]. Both drugs may facilitate earlier hospital discharge of those patients in whom stepping down to fluconazole is not an option and may overcome the limitations of currently available echinocandins which require daily intravenous administration. In addition, several other antifungal drugs for *Candida* infections are in clinical development [20], including oteseconazole which was FDA approved for vulvovaginal candidiasis.

Our study comes along with some limitations. Not all requested data were available for all patients, and the presented data reflect a real-life scenario with no predefined fungal diagnostic strategies or treatment protocols. While the geographical distribution of our samples is reflective of Europe including its laboratory capacities [24], it is still likely that settings with better access to diagnostics [25, 26] and antifungals are overrepresented. Also exact reasons for prolonged hospitalization to complete iv AF Tx were not assessed, and while some patients may have simply not been able to take oral medication, the fact that the population with prolonged hospitalization to complete iv AF Tx tended to be younger, was less severely ill and mostly on echinocandins argues against this being a major proportion of the study population. Besides resistance to fluconazole (31% of patients had isolates in the intermediate or resistant range), lack of biofilm activity an concerns about tolerability and hepatotoxicity may have been other reasons prolonged hospitalization to complete iv AF Tx. Fourthly, we did not use a standardized definition for the primary outcome of interest “prolonged hospitalization solely to complete iv AF Tx”, but relied on the treating physicians judgment. This may be considered primarily a strength, as the multiple variables factoring into the decision whether to discharge a patient are difficult to catch in a standardized definition. However, this may also be considered a weakness, as our definition is impacted by regional and national variabilities across Europe in terms of hospital length of stays, discharge criteria and opportunities for outpatient intravenous treatment, thereby limiting applicability for some hospital settings.

In conclusion, we found that across Europe ~ 1 in 7 patients (16%) with candidemia had prolonged hospitalization specifically to complete iv AF Tx underscoring that no effective oral or long acting iv alternatives are available. *C. parapsilosis* was not associated with prolonged hospitalization in contrast to *C. glabrata*, which predicted prolonged hospitalization solely to complete iv AF Tx, and which was also overrepresented among the cohort receiving outpatient iv AF Tx*.* Limitations could be overcome by new antifungals with oral formulations available, broad antifungal activity, lower rates of resistance and longer half-life. Properties, which may allow for earlier discharge and outpatient therapy reducing costs, risk for nosocomial infections and other inpatient complications.
